# Changes in Medicare Part D coverage in competitive classes in the post–Inflation Reduction Act landscape: 2024–2026

**DOI:** 10.1093/haschl/qxag171

**Published:** 2026-07-21

**Authors:** Hanke Zheng, Jonathan D Campbell, Julie A Patterson

**Affiliations:** Research Department, National Pharmaceutical Council, Washington, DC 20006, United States; Research Department, National Pharmaceutical Council, Washington, DC 20006, United States; Research Department, National Pharmaceutical Council, Washington, DC 20006, United States

**Keywords:** Inflation Reduction Act, Medicare Part D, patient access, coverage decision

## Introduction

The Inflation Reduction Act (IRA) introduced major changes to the Part D benefit design, including a cap on patient out-of-pocket costs and increased catastrophic phase liability for plans and manufacturers effective January 1, 2025. Increased plan liability may create incentives to restrict patient access by increasing formulary exclusions, utilization management, and/or patient cost-sharing.^1^ Past research reported increased Part D formulary exclusions from 2011 through 2020 broadly^2^ and for protected classes and low-tier drugs in 2025 relative to trends in 2021–2024.^3^

Incentives to increase formulary exclusions may be highest in therapeutic classes with multiple branded prescription drugs, where plans can leverage their option to exclude competitors from formularies to negotiate higher rebates. Market dynamics of increased rebates and/or rebate growth in competitive classes have previously been observed.^4,5^ This study described coverage changes of brand-only drugs in competitive classes after the Part D redesign across Medicare standalone prescription drug plans (PDPs) and Medicare Advantage Prescription Drug (MA-PD) plans, using commercial plans for comparison.

## Methods

This descriptive analysis used Fingertip Formulary (Clarivate Analytics LLC) pharmacy coverage data for Medicare and commercial plans. We evaluated coverage changes of brand-only drugs in competitive classes in MA-PD plans and PDPs from 2024 through 2026, with commercial plans serving as a control. The number of affected Medicare beneficiaries was extrapolated using 2024 enrollment data.

Competitive classes, defined as those with at least 3 commercially available, eligible, brand-only drugs, were identified based on a series of class- and drug-level inclusion and exclusion criteria (Appendix 1). Beginning with SSR Health (SSRH)–assigned classes, we excluded nondrug (eg, vaccine) classes and those with legislative coverage mandates or restrictions (eg, protected classes) or limited therapeutic relevance to older adults. We excluded classes with fewer than 3 drugs; Part D plans must generally cover at least 2 drugs in each class.^6^ We identified the US Pharmacopoeia (USP) pharmacological group or class for each remaining drug to create unique SSRH-USP class combinations that classified drugs with similar therapeutic uses (SSRH) and mechanism of action (USP). We excluded the SSRH Rare Disease class given the diversity of indications and combined diabetes drugs that were categorized across 2 USP classifications for consistency with multi-ingredient formulation matching.

We then applied drug-level exclusion criteria, excluding drugs that were physician-administered; had a commercially available independent generic or biosimilar prior to January 1, 2026; were exclusively or primarily covered by Part B; had been withdrawn or discontinued; were approved during the study period; or had a legislative mandate for coverage (ie, drugs with effective maximum fair prices in 2026). For each exclusion criteria, we used a human–dual artificial intelligence (AI) approach using Claude (Opus 4.8; Anthropic) and ChatGPT (GPT-5.5 Thinking; OpenAI). We developed standardized prompts through iterative refinement and used manual review on random samples and all instances of AI platform disagreement.

Next, we excluded any SSRH-USP class in which an otherwise-eligible drug had a first independent generic or biosimilar launch between January 1, 2024, and January 1, 2026. Because formulary data were pulled from January of each year, this removed classes where a first generic or biosimilar entry could have disrupted formulary dynamics.

We calculated changes in the percentage of beneficiaries with covered access to each drug and summarized the findings overall and by class with each insurance type. We applied paired nonparametric Wilcoxon signed-rank tests to evaluate changes from 2024 through 2026. This study followed the Strengthening the Reporting of Observational Studies in Epidemiology (STROBE) reporting guideline. This study did not involve human subjects; thus, institutional review board review was not required.

## Results

We identified 59 brand-name drugs across 16 competitive classes (Appendix 2). In 2024, the average proportion of beneficiaries with covered access was the highest in commercial plans (71.4%), followed by MA-PD plans (52.3%) and PDPs (47.4%).

Medicare coverage declined in both 2025 and 2026, with greater reductions in standalone PDPs than MA-PD plans. From 2024 to 2026, average coverage fell by 11.8 percentage points in PDPs (mean [SD]: 47.4% [0.41%] to 35.6% [0.35%]; *P* < .01) and 5.9 percentage points in MA-PD plans (52.3% [0.36%] to 46.4% [0.37%]; *P* < .01). These losses correspond to 2.7 million [M] PDP beneficiaries and 1.8M MA-PD beneficiaries losing coverage to an average included drug. Commercial coverage remained stable after the redesign (2024: 71.4%; 2026: 70.3%, *P* = .5).

At the drug level, coverage decreases from 2024 to 2026 affected over 5% of beneficiaries for over half of included drugs (50.9%; 30/59) in PDPs (representing a decline of at least 1.14 M beneficiaries per drug), 42.4% (25/59) in MA-PD plans (1.5 M beneficiaries per drug), and 20.3% (12/59) in commercial plans.

At the class level, coverage declined by at least 5 percentage points, on average, from 2024 to 2026 in 10 of 16 classes in PDPs, 7 of 16 in MA-PD plans, and in 3 included classes in commercial plans (Table 1).

**Table 1:** Average absolute percentage change of beneficiary coverage of drugs in included competitive classes from 2024 to 2026.

Drug class^a^	No.	PDPs, %	MA-PD plans, %	Commercial plans, %
ADHD	3	0.0	−0.7	0.0
COPD (combos)	6	−25.1	−5.4	−3.6
COPD (glucocorticoids)	3	8.9	13.6	2.2
Cystic fibrosis	4	−27.4	−10.0	−0.2
DMARDs (other)	3	−5.0	−2.7	1.7
Diabetes (DPP-IV inhibitors)	3	−13.2	−0.9	−6.2
Diabetes (SGLT2 inhibitors)	4	−13.1	−17.7	−7.5
Exocrine pancreatic insufficiency	3	−14.3	−1.8	2.6
Growth hormones	3	−0.6	−6.6	−3.9
HCV	3	−25.8	−0.4	−4.0
Insomnia	3	3.4	4.8	−8.6
MS	6	−21.3	−34.9	0.6
Migraine (CGRP)	6	1.3	9.2	4.9
Narcolepsy	3	−1.0	−6.6	3.3
Ophthalmics (other)	3	−32.4	−4.3	−0.4
Psoriasis	3	−8.5	−12.1	−1.7
Overall	59	−11.8	−5.9	−1.1

Abbreviations: ADHD, attention-deficit/hyperactivity disorder; CGRP, calcitonin gene–related peptide; COPD, chronic obstructive pulmonary disease; DMARD, disease-modifying antirheumatic drug; DPP-IV, dipeptidyl peptidase-4; HCV, hepatitis C virus; MA-PD, Medicare Advantage Prescription Drug; MS, multiple sclerosis; PDP, Medicare standalone Part D plan; SGLT2, sodium–glucose cotransporter 2.

^a^Drug classes are listed in the table by SSR Health (SSRH) drug class. During the class selection process, we identified unique SSRH–US Pharmacopoeia (-USP) class combinations that classified drugs with similar therapeutic uses (SSRH) and mechanism of action (USP). As a result, some SSRH classes were categorized into multiple SSRH-USP classes, with only those that met inclusion/exclusion criteria retained for the final analysis (eg, SSRH: psoriasis; USP: psoriasis agents).

## Discussion

We observed widespread and significant reductions in Medicare beneficiary access to drugs in competitive classes from 2024 to 2026, with the largest decreases in standalone PDPs. These results add to past research of increasing formulary restrictions in Medicare Part D both before and after the IRA's Part D redesign^2,3^ and support concerns around the destabilization of standalone PDPs.

This study adds to the growing literature on Part D changes in the post-IRA landscape, including increased deductibles, shifts to coinsurance, and reduced coverage of therapeutic alternatives to drugs selected to the IRA's Drug Price Negotiation Program.^1,3^ We observed greater coverage declines in PDPs despite a nearly $10 billion Centers for Medicare and Medicaid Services demonstration project intended to stabilize the PDP market, where plan offerings have decreased by half since 2024.

Commercial coverage of competitive classes remained relatively stable, suggesting that Medicare coverage declines may reflect policy impacts and/or a slowing of the rapid increases in formulary exclusions on large commercial plan formularies from 2017 to 2023.^7^ The degree to which coverage declines in Medicare reflect longer-term trends of increased formulary restrictions pre-IRA or previously reported acceleration post-IRA is not known.^2,3^ Future research with longer time horizons and statistical approaches that account for dynamic market baskets and isolate the potential impact of the IRA is needed.

Study limitations include a limited time horizon for market basket consistency and a focus on coverage as 1 measure of patient access. While commercial coverage served as a control, the study design did not allow for isolation of independent IRA effects. The number of impacted beneficiaries was extrapolated, as the Fingertip formulary captures approximately two-thirds of Medicare beneficiaries. This study did not evaluate the clinical impact of formulary exclusions, which may vary based on the total number drugs available. However, new formulary exclusions necessarily introduce nonmedical switching consistently associated with negative patient and economic outcomes.^8^ Future research is needed to evaluate changes in less-competitive classes and those where generic drugs add competitive options to brand-only drugs. Our results suggest an ongoing need to monitor Medicare patient access and outcomes and the robustness of existing Part D formulary review processes in safeguarding that access.

## Supplementary Material

qxag171_Supplementary_Data
