# Supplementary material for: Changes in Medicare Part D coverage in competitive classes in the post–Inflation Reduction Act landscape: 2024–2026
Source: Health Aff Sch. 2026 Jul 21;4(7):qxag171. doi: 10.1093/haschl/qxag171 (PMC13387062; doi:10.1093/haschl/qxag171)
Supplement: qxag171_Supplementary_Data [file qxag171_supplementary_data.zip › Supplementary Material_Clean (1).docx]

**Appendix 1. Flowchart of Competitive Class Selection**

|  | **Number of Classes** | **Number of Drugs** |
| --- | --- | --- |
| **Step 1: Class-Level Exclusions, Part 1** | 180 | 1044**^1^** |
| Protected Classes | 130 | 749 |
| Legislative Mandates (Beyond Protected Classes) or Lack of Therapeutic Relevance | 124 | 702 |
| Devices, Vaccines, Diagnostics, Imaging Agents | 120 | 661 |
| SSRH Classes with <3 Drugs | 75 | 604 |
|  |  |  |
| **Step 2: Matching SSRH-USP Classes and Class-Level Exclusions, Part 2** |  |  |
| Manual Review: Excluded Rare Diseases and Diabetes Reclassification | 74 | 543 |
| Exclude SSRH-USP Classes with < 3 Drugs | 60 | 389 |
|  |  |  |
|  |  |  |
| **Step 3: Drug-Level Criteria, Part 1** |  |  |
| Exclude Physician Administered | 57 | 315 |
| Exclude Primarily Part B | 56 | 292 |
| Exclude Withdrawn/Discontinued | 56 | 235 |
| Exclude Drugs Approved After 1/1/2024 | 56 | 233 |
| Exclude Generic Available by 1/1/2026 | 41 | 125 |
| Exclude Drugs with a Legislative Mandate for Coverage | 40 | 121 |
|  |  |  |
| **Step 4: Class-Level Exclusions, Part 3** |  |  |
| Exclude Generic Launch within SSRH-USP Class Combination, 1/1/2024-1/1/2026 | 35 | 105 |
| Exclude SSRH-USP Classes with < 3 Drugs | 16 | 58 |
|  |  |  |
| **Step 5: Drug-Level Criteria, Part 2** |  |  |
| Separate Remaining Combination and Single-Ingredient Products^2^ | 16 | 59 |

^1^The starting 1044 drugs represent the entire sample downloaded from SSR Health.

^2^ Brand-name drugs grouped by SSR Health were separated and analyzed as distinct drugs.

**Appendix 2. Identified Drugs in Competitive Classes**

| **Generic Name** | **Drug Class*** |
| --- | --- |
| methylphenidate | ADHD |
| methylphenidate hydrochloride | ADHD |
| viloxazine hydrochloride | ADHD |
| umeclidinium bromide; vilanterol trifenatate | COPD (Combos) |
| glycopyrrolate; formoterol fumarate | COPD (Combos) |
| fluticasone furoate; vilanterol trifenatate | COPD (Combos) |
| budesonide; glycopyrrolate; formoterol fumarate | COPD (Combos) |
| formoterol fumarate; mometasone furoate | COPD (Combos) |
| fluticasone furoate; umeclidinium bromide; vilanterol trifenatate | COPD (Combos) |
| ciclesonide | COPD (Glucocorticoids) |
| fluticasone furoate | COPD (Glucocorticoids) |
| mometasone furoate | COPD (Glucocorticoids) |
| ivacaftor | Cystic Fibrosis |
| lumacaftor; ivacaftor | Cystic Fibrosis |
| tezacaftor; ivacaftor | Cystic Fibrosis |
| elexacaftor; tezacaftor; ivacaftor | Cystic Fibrosis |
| sitagliptin phosphate; metformin hydrochloride | Diabetes (DPP-4 Inhibitors) |
| linagliptin; metformin hydrochloride | Diabetes (DPP-4 Inhibitors) |
| linagliptin | Diabetes (DPP-4 Inhibitors) |
| canagliflozin; metformin hydrochloride | Diabetes (SGLT2 Inhibitors) |
| canagliflozin | Diabetes (SGLT2 Inhibitors) |
| empagliflozin; metformin hydrochloride | Diabetes (SGLT2 Inhibitors) |
| dapagliflozin; metformin hydrochloride extended-release | Diabetes (SGLT2 Inhibitors) |
| baricitinib | DMARDs (Other) |
| upadacitinib | DMARDs (Other) |
| tofacitinib citrate | DMARDs (Other) |
| pancrelipase^ | Exocrine Pancreatic Insufficiency |
| pancrelipase | Exocrine Pancreatic Insufficiency |
| pancrelipase | Exocrine Pancreatic Insufficiency |
| somatropin | Growth Hormones |
| somatropin | Growth Hormones |
| somatropin | Growth Hormones |
| glecaprevir; pibrentasvir | HCV |
| sofosbuvir | HCV |
| sofosbuvir; velpatasvir; voxilaprevir | HCV |
| suvorexant | Insomnia |
| lemborexant | Insomnia |
| daridorexant hydrochloride | Insomnia |
| erenumab-aooe | Migraine (CGRP) |
| fremanezumab-vfrm | Migraine (CGRP) |
| galcanezumab-gnlm | Migraine (CGRP) |
| rimegepant sulfate | Migraine (CGRP) |
| atogepant | Migraine (CGRP) |
| ubrogepant | Migraine (CGRP) |
| interferon beta-1a | MS |
| interferon beta-1b | MS |
| siponimod fumarate | MS |
| peginterferon beta-1a | MS |
| interferon beta-1a | MS |
| diroximel fumarate | MS |
| solriamfetol hydrochloride | Narcolepsy |
| pitolisant hydrochloride | Narcolepsy |
| calcium oxybate; magnesium oxybate; potassium oxybate; sodium oxybate | Narcolepsy |
| satralizumab-mwge | Ophthalmics (Other) |
| varenicline tartrate | Ophthalmics (Other) |
| lifitegrast | Ophthalmics (Other) |
| halobetasol propionate; tazarotene | Psoriasis |
| apremilast | Psoriasis |
| tapinarof | Psoriasis |

*Drug classes are listed in the Table by SSR Health drug class. During the class selection process, we identified unique SSRH-US Pharmacopoeia (USP) class combinations that classified drugs with similar therapeutic uses (SSRH) and mechanism of action (USP). As a result, some SSRH classes were split into multiple SSRH-USP classes, with only those that met inclusion/exclusion criteria retained for the final analysis (e.g., SSRH: Psoriasis; USP: Psoriasis Agents).

^A generic name appearing multiple times reflects the inclusion of distinct brand-name products that share that generic name.
